# Echocardiographic Findings in Heart Failure Patients With Methamphetamine Use: A Case-Control Study

**DOI:** 10.7759/cureus.16170

**Published:** 2021-07-04

**Authors:** Roopam Jariwal, Vishal Narang, Nadia Raza, Baldeep Mann, Janpreet Bhandohal, Michael Valdez, Theingi Tiffany Win, Fowrooz S Joolhar, Aslan Ghandforoush

**Affiliations:** 1 Internal Medicine, University of California, Los Angeles-Kern Medical Center, Bakersfield, USA; 2 Cardiology, University of California, Los Angeles-Kern Medical Center, Bakersfield, USA

**Keywords:** methamphetamine induced cardiomyopathy, heart failure with reduced ejection fraction, diastolic dysfunction, transoesophageal echo, echo

## Abstract

Background

Methamphetamine use is associated with cardiovascular disease and significant morbidity and mortality. There is only one previous study performed on echocardiographic parameters in patients with methamphetamine cardiomyopathy.

Methods

We performed a retrospective review of medical records in a county hospital in Southern California with a high population of methamphetamine users. We reviewed medical records and echocardiogram findings in patients seen in our institution from November 2019 to November 2020 who had cardiomyopathy with and without methamphetamine use. We excluded patients who either left the hospital or expired before appropriate assessment. We divided our patient population into a case group (methamphetamine users) and a control group (non-methamphetamine users) to study and compare their echocardiographic parameters.

Results

Case group included a total of 254 patients and control group included 268 patients. Majority of the patient population were males - 178 (70%) and 180 (67%) in the case and control group respectively. Age was found to be statistically significant with the younger population in the case group (p = 0.0000). Our analysis revealed statistically significant difference in methamphetamine users compared to non-users in regards to left ventricle ejection fraction (33.65% ± 18.02 vs. 41.55% ± 15.61, p=0.0000), left ventricle mass index (122.49 grams/m^2^ ± 40.66 vs. 108.62 grams/m^2^ ± 32.82, p=0.0000), left ventricle end diastolic volume index (85.91 mL/m^2^ ± 37.40 vs. 72.44 mL/m^2^ ± 25.44; p=0.0000) and marginally significant right ventricle systolic pressure (42.29mmHg ± 17.53 vs. 39.59mmHg ± 15.61; p=0.0540)

Conclusion

Our results indicated that methamphetamine users had echocardiogram findings with decreased ejection fraction and increased left ventricular mass index, end-diastolic volume index, and right ventricular systolic pressure consistent with worse dilated cardiomyopathy comparison to non-users.

## Introduction

Methamphetamine activates the sympathetic system, resulting in tachycardia, hypertension, vasospasm/vasoconstriction, and myocardial wall stress or ischemia [1]. The exact prevalence of methamphetamine associated cardiomyopathy is unknown; however, the prevalence is on an incline due to increased drug usage. An echocardiogram is a noninvasive test commonly used to assess myocardial and valvular structure, quantify chamber size, and estimate ejection fraction. Additional specific details such as wall motion, wall thickness, and chamber size have been correlated to clinical conditions. Methamphetamine associated cardiomyopathy has been reported to have typical echocardiographic findings of dilated cardiomyopathy with reduced left ventricular systolic function and cardiac chamber enlargement [1]. Many overlapping features may be seen on echocardiograms from the various etiologies of cardiomyopathy, such as ischemic heart disease [2]. Assessments for wall motion abnormalities, cardiac chamber enlargement, and decline in left ventricular ejection fraction provide information and prognosis about an individual patient's clinical condition. Typical factors attributed to influence left ventricle mass index (LVMI) include body size, ethnicity, and exercise-related factors. However, LVMI has been shown to predict cardiovascular events and premature death independently [3,4]. Other parameters such as left ventricular end-diastolic volume (LV EDV) indicate left ventricular function and are standardized to a body-surface-area ratio. It represents the volume of blood at the end load filling and can be quantified via echocardiogram. Depending on the value, it may indicate enlargement compared to a normal value of <82 ml/m^2^ [5]. The right ventricle is designed to deliver a venous return to a low-pressure system. Right ventricular systolic pressure (RVSP) has been adopted as a marker to evaluate for pulmonary hypertension. It is equivalent to pulmonary artery systolic pressure in the absence of pulmonary outflow tract obstruction and is associated with reduced survival in patients with heart disease [6]. The purpose of our study was to characterize various echocardiographic findings, including ejection fraction, right ventricular systolic pressure, cardiac mass index, and left ventricular end-diastolic volume amongst heart failure patients with and without a history of methamphetamine use.

## Materials and methods

We intended to perform a case-control study in heart failure patients (HF) patients with active methamphetamine usage compared to patients with cardiomyopathy without methamphetamine use. We observed the echocardiographic characteristics in both patient populations. The primary aim of this study is to determine whether there is any significant difference in echocardiographic parameters in heart failure patients with a history of methamphetamine use that would potentially determine their prognosis compared to patients without methamphetamine use. We included Right Ventricular Systolic Pressure (RVSP), Left Ventricular Ejection Fraction (LVEF), Left Ventricular Mass Index (LVMI), End Diastolic Volume Index (EDVI), grade of Diastolic Dysfunction (DD) along with age and gender to determine if there is any statistical difference between the two groups. The ejection fraction was calculated using the Modified Simpson Method (biplane method of disks).

**Figure 1:**
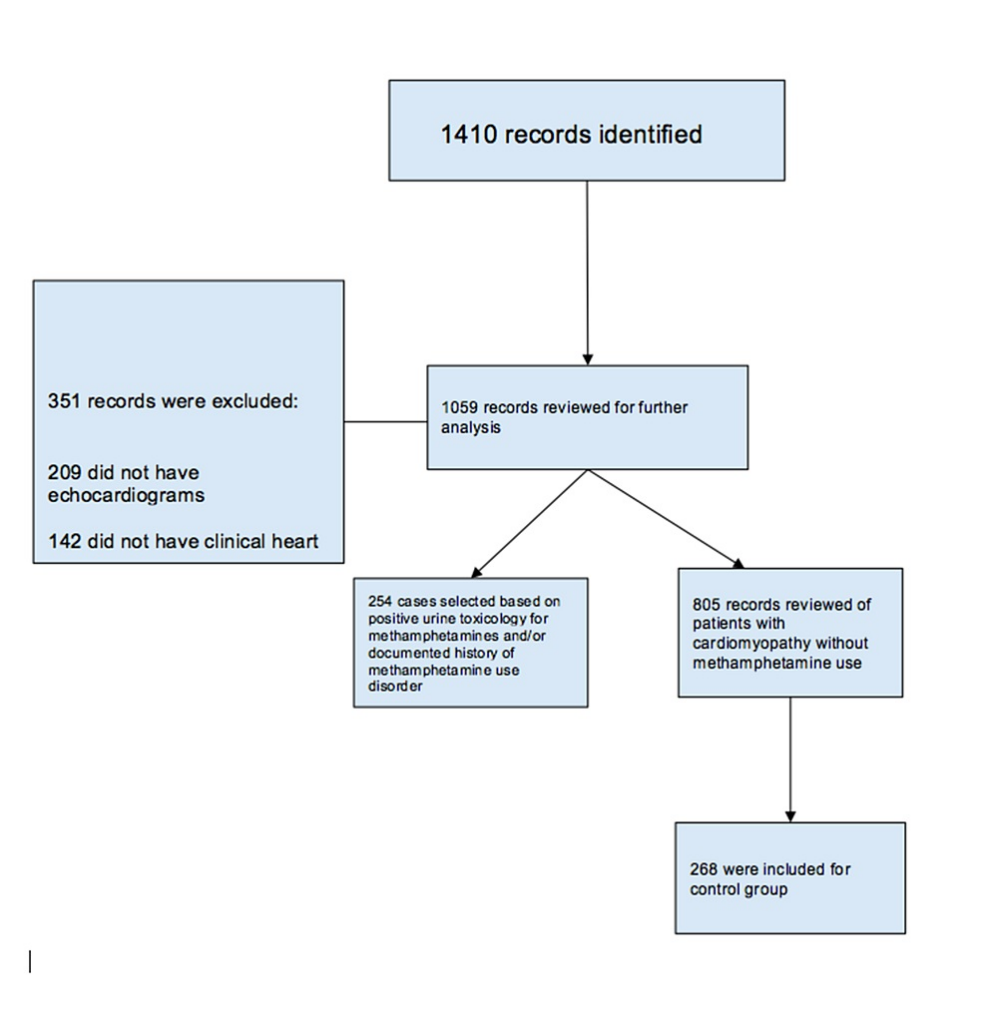
Selection criteria for the study

After obtaining institutional review board (IRB) approval, we performed a retrospective chart review. We screened patient charts from November 1, 2019, to November 1, 2020, using heart failure related International Classification of Diseases, Tenth Revision, Clinical Modification (ICD-10 CM) codes that showed 1410 records. Our inclusion criteria included patients aged 18 years and above with clinical heart failure and active and recent methamphetamine use (in the last six months) based on history provided or urine toxicology, along with patients who underwent echocardiography during the index admission. We excluded patients who either left the hospital or expired before an echocardiogram was obtained. The final case group included 254 patients (Figure 1). Controls were screened using the same criteria except that patients were negative for methamphetamine on urine toxicology and history. Charts were also reviewed for a history of methamphetamine use. Patients were included in the case group if they were actively using methamphetamine based on history, even if urine toxicology was negative.

Data was collected for index admission, defined as the first admission to our institution for heart failure symptoms. The parameters were taken from echocardiography that was performed in our institution. The results for RVSP, LVMI, EDVI are reported as a continuous numerical value. As for LVEF, it is either reported as an exact numerical value or as a range within five value points. As a result, LVEF was rounded off to the nearest numerical value or an average value was taken for the range as follows: LVEF of <10 was entered as 10; 10-15 as 12; 15-20 as 17; 20-25 as 23; 25-30 as 27; 30-35 as 33; 35-40 as 37; 40-45 as 43; 45-50 as 47; 50-55 as 52; 55-60 as 58. Diastolic dysfunction is reported as grade 1 to 3. Grade 0 was used for patients who had no diastolic dysfunction.

All the data was compiled into an excel sheet. Patient identifiers were not disclosed except to the members of the data collection team. Data were analyzed using R version 4.0.0. A two-sample one-tailed t-test was used for RVSP, LVEF, LVMI, EDVI and age. Odds ratio and chi-square test of independence were used for diastolic dysfunction. Subgroup analyses were performed for parameters where the values were not available for all the patients.

## Results

The final case group included 254 patients after the application of inclusion and exclusion criteria. The final control group included 268 patients. Majority were males, 178 (70%) and 180 (67%) in the case and control group respectively which was not statistically significant (odds ratio = 1.1450, p = 0.4735) (Table 1). The results for RVSP, LVEF, LVMI, EDVI and age are based on a two-sample one-tailed t-test, whereas diastolic dysfunction was reported using odds ratio and chi-squared test of independence.

**Table 1:** Case/Control by Gender

	Male (n)	Female (n)	Odds ratio (95% CI); p-value
Case	178	76	1.1450 (0.7907,1.6582); 0.4735
Control	180	88	

**Figure 2:**
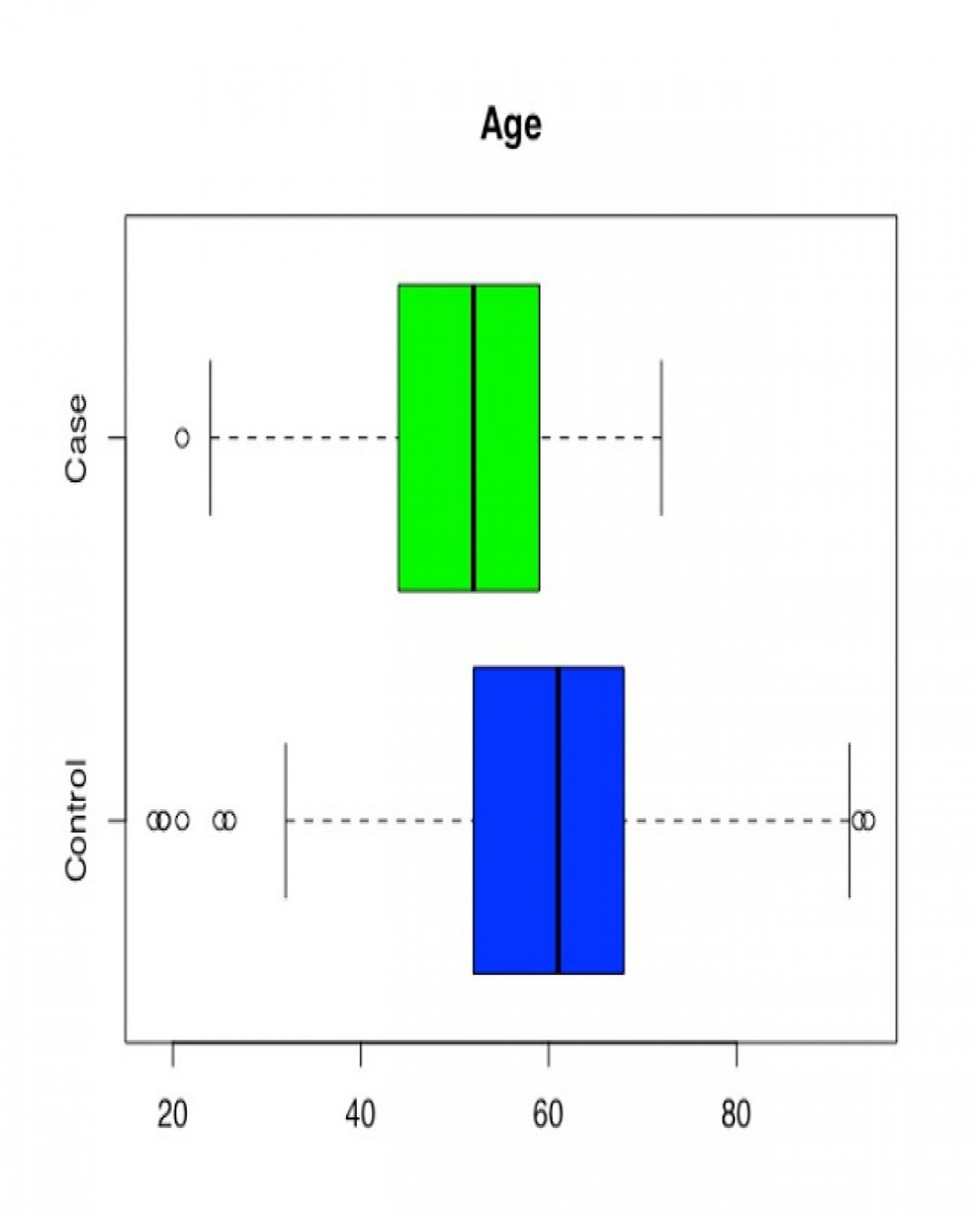
Box and Whisker plot for Case/Control by Age

**Figure 3:**
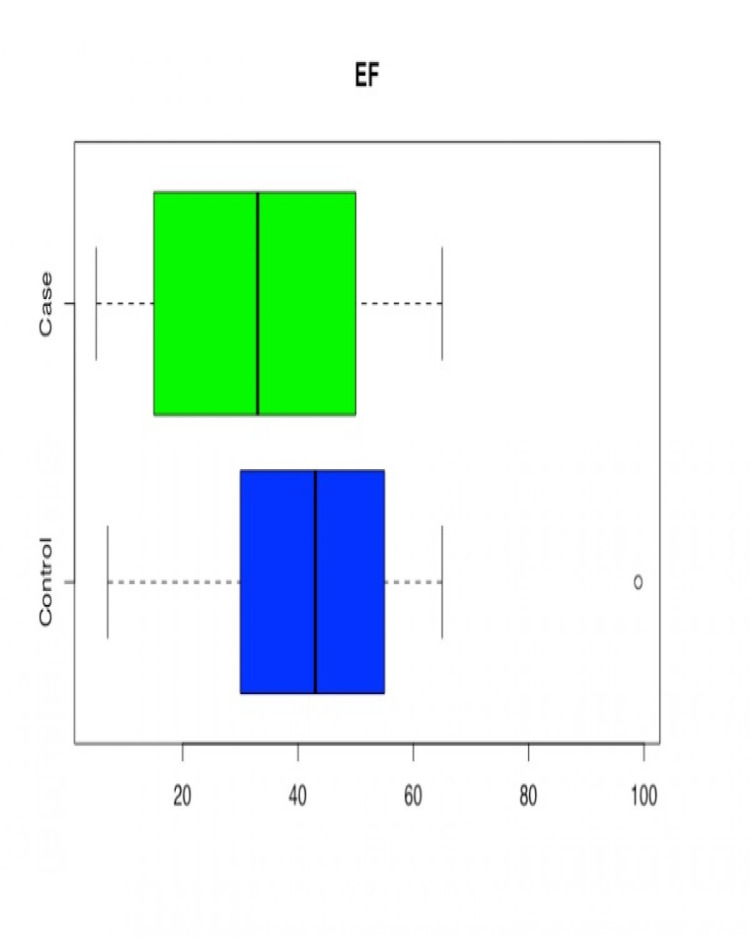
Box and Whisker plot for Ejection Fraction (EF) in Case/Control Groups

**Figure 4:**
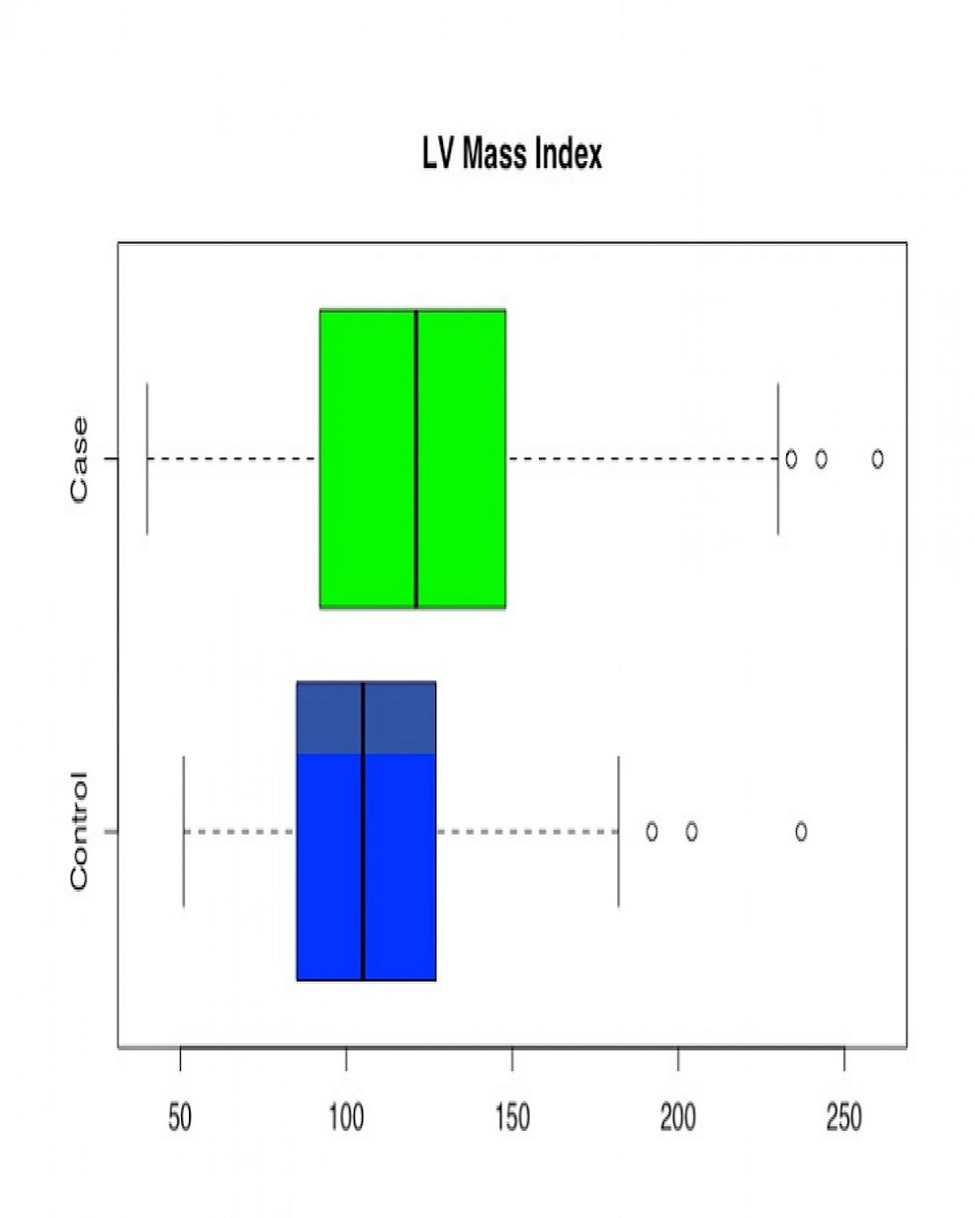
Box and Whisker plot for Left Ventricular Mass Index (LVMI) for Case/Control groups

**Figure 5:**
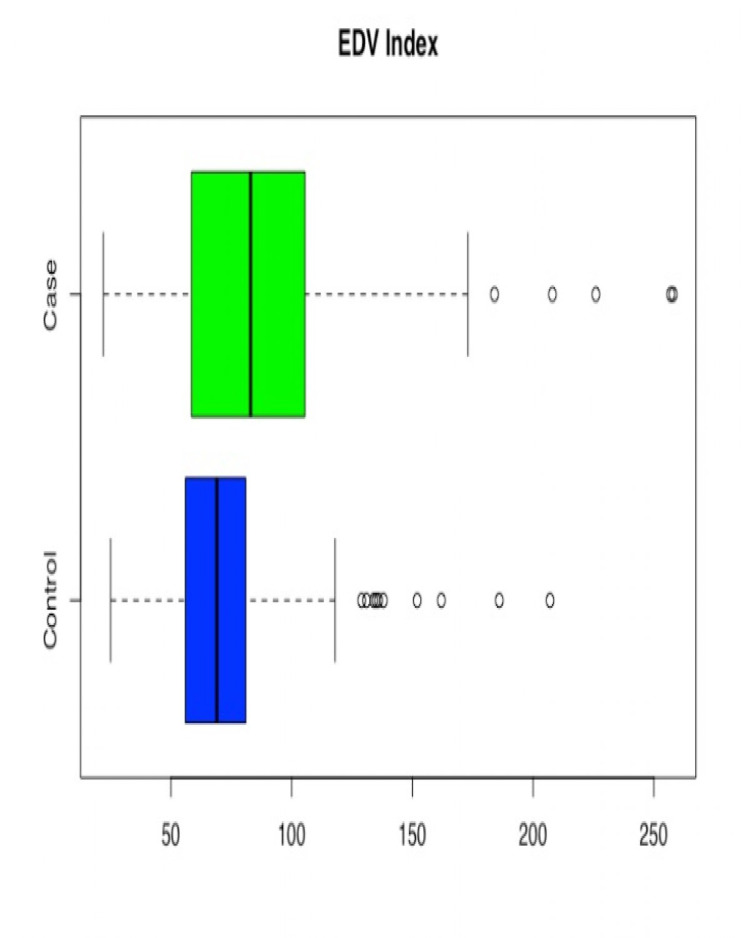
Box and Whisker plot for End Diastolic Volume Index (EDVI) for Case/Control groups

**Figure 6:**
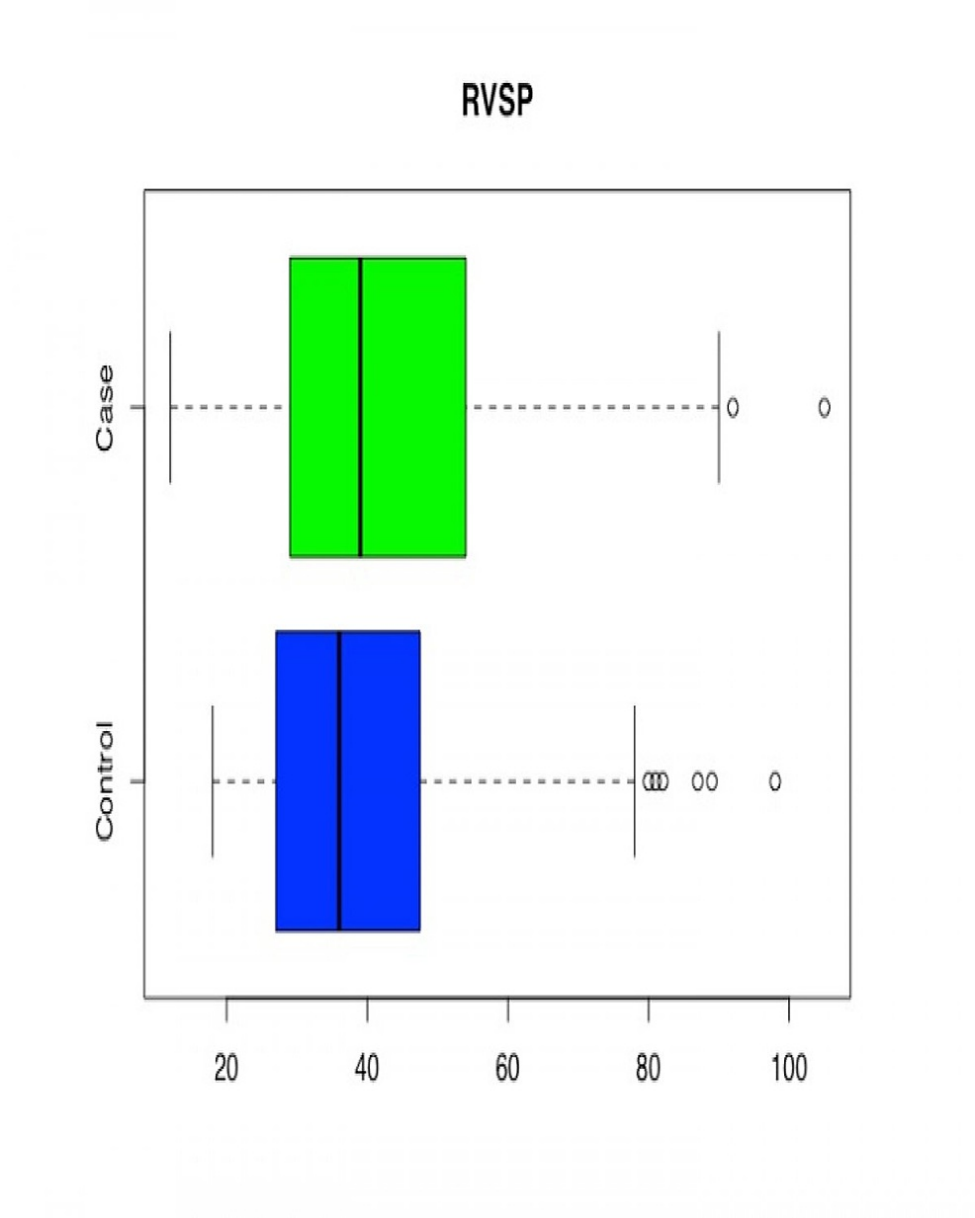
Box and Whisker Plot for Right Ventricular Systolic Pressure (RVSP) for Case/Control groups

Age was found to be statistically significant with the younger population in the case group (p = 0.0000). The mean value LVEF in the control group was higher than in the case group (p= 0.0000), suggesting better left ventricular systolic function in patients without methamphetamine use. LVMI, which signifies the mass of left ventricle as per body surface area, was significantly higher in the case group (p= 0.0000). So was the EDV Index (p=0.0000), signifying greater left ventricular dilatation in methamphetamine users. In patients where RVSP value was available (179 patients, 211 controls), mild statistical significance (p-value = 0.0540) was observed in the difference in RVSP between case and control groups (cases having higher RVSP). Given that not all the patients had RVSP reported, results may have been impacted by a smaller sample size (Table 2, Figures 2-6).

**Table 2:** Case/Control by Age Abbreviations: Right Ventricular Systolic Pressure (RVSP), Left Ventricular Ejection Fraction (LVEF), Left Ventricular Mass Index (LVMI), End Diastolic Volume Index (EDVI), SD: Standard Deviation, SE: Standard Error, n: sample size

	n	Mean ± SD	Median	SE	p-value
Age (Years)					0.0000
Case	254	50.74 ± 10.37	52	0.6507	
Control	268	59.88 ± 13.97	61	0.8534	
LVEF (%)					0.0000
Case	251	33.65 ± 18.02	33	1.1374	
Control	264	41.55 ± 15.61	43	0.9607	
LV Mass Index (grams/m^2^)					0.0000
Case	190	122.49 ± 40.66	121	2.9498	
Control	213	108.62 ± 32.82	105	2.2488	
EDV Index (mL/m^2^)					0.0000
Case	243	85.91 ± 37.40	83	2.3992	
Control	257	72.44 ± 25.44	69	1.5869	
RVSP (mmHg)					0.0540
Case	179	42.29 ± 17.53	39	1.3103	
Control	211	39.59 ± 15.61	36	1.0746	

While comparing diastolic dysfunction between cases (32/129, 24.81%) and controls (35/134, 26.12%) where dysfunction was defined as any grade (1, 2 or 3); the odds ratio was 1.07, not statistically significant (p = 0.807) which means that the incidence of diastolic dysfunction was similar in both groups. Diastolic dysfunction was also broken down as per the grade, which again showed no statistical significance (p = 0.77), demonstrating similar incidence between two groups regarding different dysfunction grades. However, the sample size was smaller in individual grade groups (Table 3).

**Table 3:** Case/Control by Diastolic Dysfunction

Diastolic dysfunction	Cases (n)	Controls (n)	Odds ratio (95% CI); p-value
None (0)	32	35	1.07 (0.6151,1.8671); 0.807
Any level (1/2/3)	97	99	
			p-value
None (0)	32	35	0.77
Grade 1	30	30	
Grade 2	41	48	
Grade 3	26	21	

## Discussion

**Figure 7:**
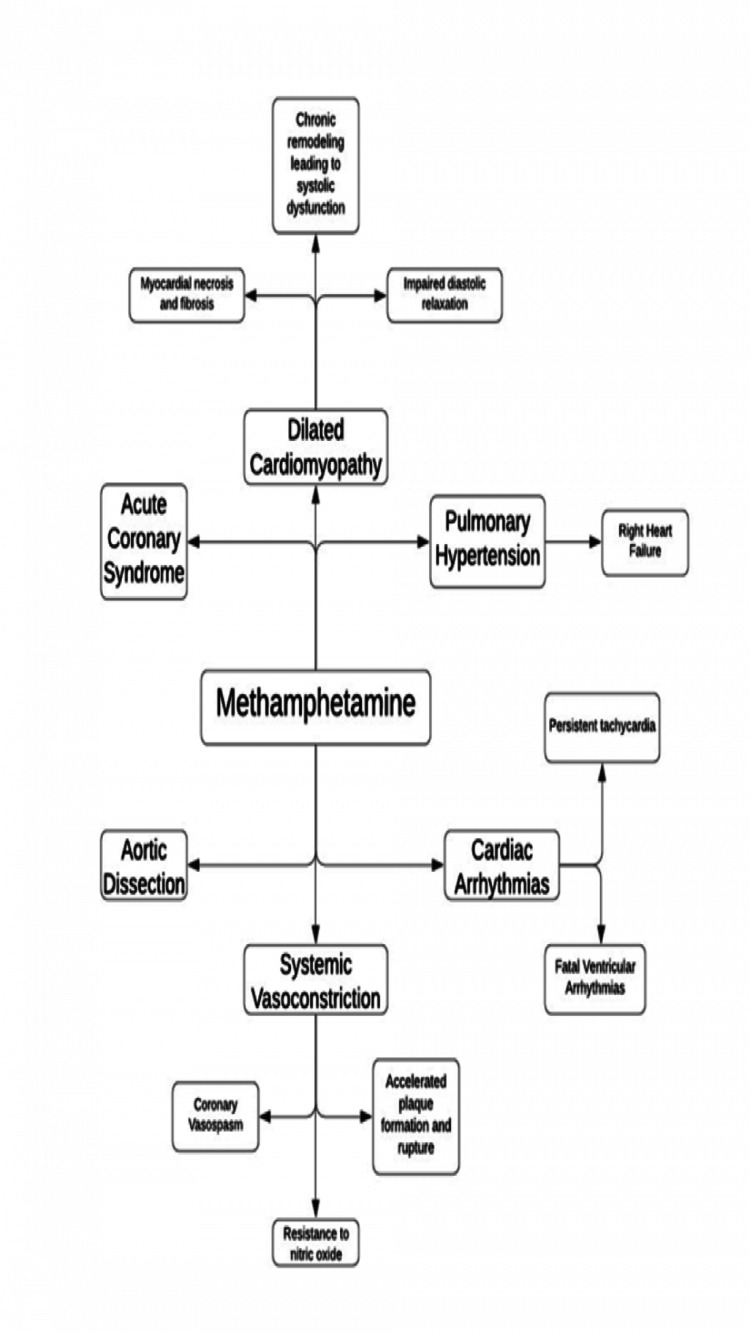
Cardiovascular Manifestations of Methamphetamine

Methamphetamine is a highly potent and addictive substance that was first created in the late 1920s to mimic the nasal vasoconstrictor ephedrine. The addition of the "methyl" group to amphetamines further enhanced its potency by making it lipophilic and its ability to penetrate the blood-brain barrier. In chronic methamphetamine users, the drug permanently alters the user's neurological function by affecting cognitive, psychiatric, and behavioural modalities. Similarly, methamphetamine users have various cardiovascular manifestations that include but are not limited to accelerated coronary plaque formation, cardiac arrhythmias, pulmonary hypertension, coronary vasospasm, and cardiac remodelling leading to cardiomyopathy, hypertension, aortic dissection, and acute coronary syndromes (Figure 7). Due to this, cardiovascular disease is the second leading cause of death in methamphetamine users after overdose [7].

Although methamphetamine consumption occurs independent of socioeconomic status, geographics, race, and culture, most consumers tend to be younger. The cardiac complications of methamphetamine are hypothesized to arise from a variety of mechanisms. In human autopsy specimens, severe interstitial fibrosis and scar formation has been documented [8]. Physiological pathways implicated include catecholamine surges acutely during methamphetamine intoxication with contingent hypertensive crises, longer-term upregulation of the sympathetic axis, and myocardial toxicity with impaired cellular metabolism. Depending on which process predominates, different patterns of pathology may develop, particularly in the case of methamphetamine-associated cardiomyopathy [9].

Preclinical studies have also shown that methamphetamine-induced endothelial nitric oxide synthase activation and endothelin‐1 release leads to potent vasoconstriction [10]. Further, chronic methamphetamine users are less responsive to the vasodilatation effects of nitroglycerin [11].

Prolonged use of methamphetamine, especially when used intravenously, directly accumulates in the lungs. It is then hypothesized to be engulfed by pneumocytes leading to a similar surge of radical oxygen species formation, causing endothelial damage and pulmonary hypertension. Methamphetamine users are at a 27% increased risk of sudden cardiac death, as reported by the United States National Inpatient Sample database that studied over 180,000 methamphetamine drug users. Autopsies obtained from chronic methamphetamine users showed cardiac fibrosis and necrosis findings that were directly proportional to the duration and frequency of drug use. These chronic structural changes likely increased the risk of ventricular arrhythmia due to electrical conduction abnormalities of the heart [1,7].

Methamphetamine causes systolic dysfunction by causing chronic remodelling and left ventricular dilatation. In a study performed by Yu et al., mice were administered large doses of methamphetamine and 12 weeks later were found to have concentric left ventricular hypertrophy, myocardial fibrosis, necrosis and decreased contractile capacity [12]. In contrast, in an additional study performed by Lord et al., echocardiographic parameters were studied in mice given binge doses of methamphetamine. This study showed left ventricular dilatation with contractility and relaxation dysfunction - and, subsequently, an increase in left ventricular volume and a decrease in left ventricular wall thickness leading to eccentric dilated cardiomyopathy. Impaired relaxation suggested diastolic dysfunction with echocardiogram findings of increased end-diastolic volume and pressure [13].

A similar study performed in Germany by Schurer et al. studied thirty endomyocardial biopsies of methamphetamine users to evaluate their overall outcome with or without cessation of methamphetamine, the extent of fibrosis affecting clinical symptoms, and echocardiographic features. Their results were significant for the echocardiographic prevalence of impaired ejection fraction, increase in left ventricular end-diastolic diameter, with improvement in these factors with discontinuation of the drug [14].

To the best of our knowledge, there is only one previous study before ours that studied these specific echocardiogram features in methamphetamine users: ejection fraction (EF), end-diastolic volume (EDV), left ventricle mass index (LVMI), right ventricle systolic pressure (RVSP). In this study performed by Ito et al., in Hawaii in 2009, 28 methamphetamine users (case group) were compared to non-users revealing a statistically significant decrease in LVEF, increase in LV EDV; however, the results for LVMI and RVSP were not statistically significant. This possibly was due to their small sample size. Similar to cocaine, methamphetamine is hypothesized to activate the calcium/calmodulin-dependent protein kinase pathway that causes myocardial fibrosis and necrosis; methamphetamine likely cause the same effect physiologically. Though this study did not show a statistically significant increase in LVMI, they acknowledged their limitations due to the small sample size. Their results were still consistent with autopsy studies performed in San Francisco, California, that supported evidence of methamphetamine users to have enlarged hearts that weighed more than their age-matched controls. Our study showed statistically significant results for an increase in LVMI in methamphetamine users, which we hypothesize due to a chronic burden of hypertension and tachycardia leading to LV hypertrophy. The exact mechanism of myocardial injury remains unknown; however, a combination of LV hypertrophy due to significant vasoconstriction increased myocardial afterload and myocardial necrosis, fibrosis leading to eccentric dilatation and impaired relaxation causes this distinctive catastrophic heart failure [15].

Cocaine abuse causes higher LVMI, and lower EF [16,17]. Methamphetamine like cocaine has sympathomimetic effects causing vasoconstriction of coronary arteries, tachycardia, and direct myocardial toxicity; both may also have similar pathophysiological effects on the myocardium. Indeed, the sympathomimetic and toxic effects on the heart induced by methamphetamine are likely worse than cocaine because it increases catecholamine release rather than just inhibiting its reuptake [18].

Our study is limited by several factors but mainly by the inclusion of only select echocardiographic parameters. We also did not include any demographic data other than age and gender. The small sample size also limits our study as we only looked into patients' data spanning one year (from 2019 to 2020) due to a change in our electronic medical record database before the year 2019. Our case group included patients with heart failure that had recent or active methamphetamine usage as correlating factor, not necessarily as the only causation of heart failure.

## Conclusions

In conclusion, patients with methamphetamine-related cardiomyopathy tend to be younger and had worse cardiomyopathy related echocardiogram parameters when compared to patients with cardiomyopathy without methamphetamine use. Methamphetamine users had increased LV mass index, worse systolic dysfunction and increased chances of developing right heart failure from increased overall RVSP. Further large scale, randomized controlled trials are needed to confirm these findings of methamphetamine-related cardiomyopathy.
